# Supplementary material for: TNF and type I IFN induction of the IRG1-itaconate pathway restricts Coxiella burnetii replication within mouse macrophages
Source: bioRxiv. 2023 Jul 7:2023.07.07.548079. Preprint. [Version 1] doi: 10.1101/2023.07.07.548079 (PMC10350068; doi:10.1101/2023.07.07.548079)

bioRxiv preprint doi: https://doi.org/10.1101/2023.07.07.548079; this version posted July 7, 2023. The copyright holder for this preprint (which was not certified by peer review) is the author/funder, who has granted bioRxiv a license to display the preprint in perpetuity. It is made available under aCC-BY 4.0 International license.

Supplemental Figures

Figure S1. related to Figure 1. T4SS is required for *C. burnetii* translocation of CBU_0077 into murine BMDMs. C57BL/6J (WT), *Tnfr1*$^{-/-}$, and *Tnfr2*$^{-/-}$ BMDMs, with and without 10 ng/ml rTNF, were infected with BlaM-CBU_0077-expressing *icmL:Tn C. burnetii* at an MOI of 500. At 24 hours post-infection, BMDMs were loaded with CCF4-AM and analyzed by flow cytometry. Shown are representative flow cytometric plots. Bar graph depicting percent injection is showing the combined data from five independent experiments.

Figure S2. related to Figure 2. TNF restricts *C. burnetii* T4SS injection in murine macrophages independently of ROS and RNS production and RIPK3- and caspase-8-mediated cell death. C57BL/6J (WT), *Nos2*$^{-/-}$, *Gp91phox*$^{-/-}$, and *Ripk3*$^{-/-}$*Casp8*$^{-/-}$ BMDMs were infected with BlaM-CBU_0077-expressing WT or *icmL:Tn C. burnetii* at an MOI of 500. At 24 hours post-infection, BMDMs were loaded with CCF4-AM and analyzed by flow cytometry. Flow cytometric plots are representative of three independent experiments.

Figure S3. related to Figure 4. siRNA-mediated silencing of *Irg1* and fluorescent imaging of murine BMDMs infected with *C. burnetii*. (A) C57BL/6 (WT) BMDMs were stimulated overnight with 10 ng/ml rTNF and transfected with control scrambled or *Irg1*-targeting siRNA. They were then infected with mCherry-expressing WT *C. burnetii* at an MOI of 50. *Irg1* knockdown efficiency was analyzed at Day 0 and Day 7 by by qPCR. Bar graphs show *Irg1* mRNA expression relative to the housekeeping gene *Gapdh*. Representative graph of 4 independent experiments. (B) Representative fluorescence micrographs of infected WT BMDMs treated with control scrambled or *Irg1* siRNA treatment. BMDMs were fixed and stained with DAPI on Day 7 post-infection and imaged at 40X magnification.

Figure S4. related to Figure 5. Itaconic acid restricts *C. burnetii* intracellular growth. WT BMDMs were treated with or without 1mM or 10mM itaconic acid (ITA) and were then infected with mCherry-expressing WT *C. burnetii* at an MOI of 50. ITA was added or removed at 24 and 72 hours post-infection. Bacterial uptake and replication were measure respectively at Day 0 and Day 7 by measuring genomic equivalents (GE) by qPCR. Bar graphs show fold change relative to Day 0 GE levels ± SEM. Shown are the combined data from 3 independent experiment with triplicate wells.
.

bioRxiv preprint doi: https://doi.org/10.1101/2023.07.07.548079; this version posted July 7, 2023. The copyright holder for this preprint (which was not certified by peer review) is the author/funder, who has granted bioRxiv a license to display the preprint in perpetuity. It is made available under aCC-BY 4.0 International license.

Supplemental Figure 1

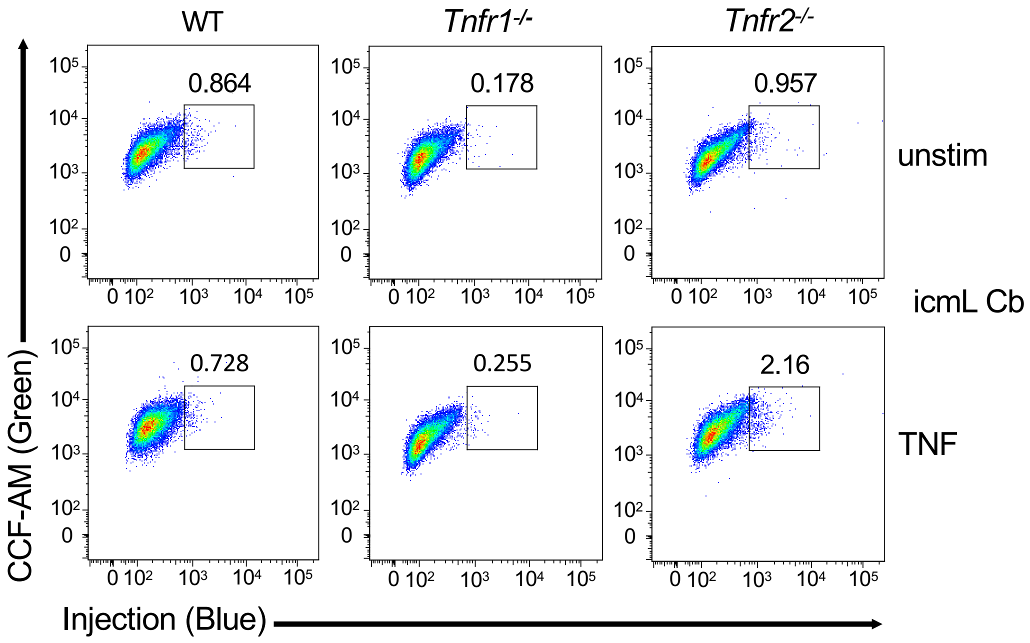

bioRxiv preprint doi: https://doi.org/10.1101/2023.07.07.548079; this version posted July 7, 2023. The copyright holder for this preprint (which was not certified by peer review) is the author/funder, who has granted bioRxiv a license to display the preprint in perpetuity. It is made available under aCC-BY 4.0 International license.

Supplemental Figure 2

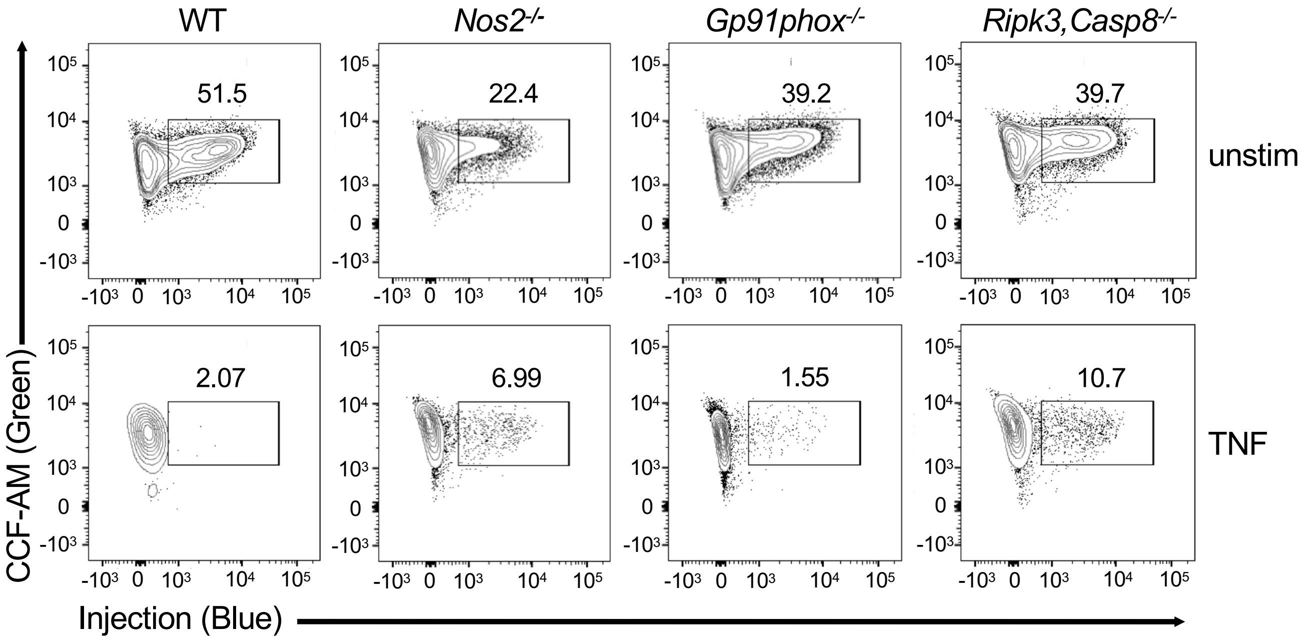

bioRxiv preprint doi: https://doi.org/10.1101/2023.07.07.548079; this version posted July 7, 2023. The copyright holder for this preprint (which was not certified by peer review) is the author/funder, who has granted bioRxiv a license to display the preprint in perpetuity. It is made available under aCC-BY 4.0 International license.

Supplemental Figure 3

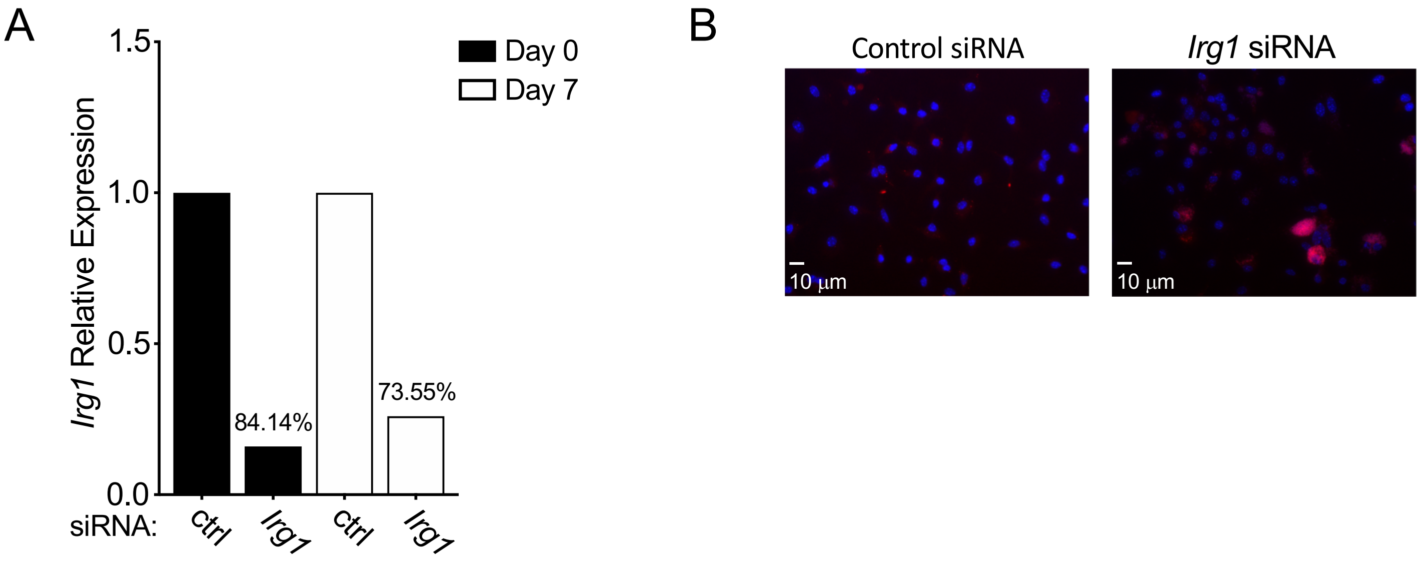

bioRxiv preprint doi: https://doi.org/10.1101/2023.07.07.548079; this version posted July 7, 2023. The copyright holder for this preprint (which was not certified by peer review) is the author/funder, who has granted bioRxiv a license to display the preprint in perpetuity. It is made available under aCC-BY 4.0 International license.

Supplemental Figure 4

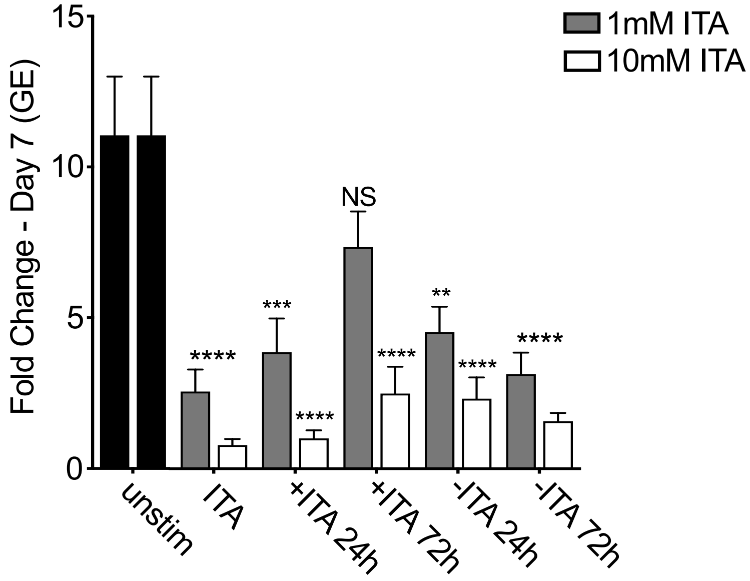

Supplement: Supplement 1 [file NIHPP2023.07.07.548079v1-supplement-1.pdf]
